# Apple metabolism under oxidative stress affects plant cell wall structure and mechanical properties

**DOI:** 10.1038/s41598-023-40782-6

**Published:** 2023-08-24

**Authors:** Piotr Mariusz Pieczywek, Agata Leszczuk, Magdalena Kurzyna-Szklarek, Justyna Cybulska, Zbigniew Jóźwiak, Krzysztof Rutkowski, Artur Zdunek

**Affiliations:** 1 Institute of Agrophysics Polish Academy of Sciences, Lublin, Poland; 2 Institute of Horticulture - National Research Institute, Skierniewice, Poland

**Keywords:** Plant stress responses, Atomic force microscopy, Immunological techniques

## Abstract

Several studies have shown beneficial effects of short exposure to oxidative stress on stored fruit, such as better preservation, increased firmness, preservation of polyphenolic compounds, and reduced risk of postharvest disorders such as bitter pit and superficial scald in apples. In this study the effect of short-term oxidative stress conditions on the physiology of apple fruit was investigated. Apple fruit of three cultivars were exposed to hypoxic storage conditions of various lengths to induce anaerobiosis. The response of apple fruit to short-term oxidative stress was evaluated by means of cell wall immunolabeling and atomic force microscopy. In addition, the antioxidant capacity and antioxidative activity of apple peels was assessed. Through various techniques, it was shown that short-term oxidative stress conditions promote specific enzymatic activity that induces changes in the cell wall of apple fruit cells. Exposure to short-term stress resulted in the remodeling of cell wall pectic polysaccharides, observed as an increase in the size and complexity of extracted oxalate pectin. Structural changes in the cell wall were followed by an increase in Young’s modulus (compressive stiffness of a solid material, expressed as the relationship between stress and axial strain) of the cell wall material. The data presented in this paper show in a novel way how storage under short-term oxidative stress modifies the cell wall of apple fruit at the molecular level.

## Introduction

The most common technique for the post-harvest storage of apples is the use of a controlled atmosphere (CA). Reduced oxygen content, high humidity and low temperature slow down fruit metabolic processes related to respiration and ethylene production, enabling high product quality to be maintained. However, long-term storage inevitably raises the risk of the fruit developing physiological disorders leading to significant drops in quality and the loss of large batches of apples. New techniques such as ultra-low oxygen (ULO) and dynamically controlled atmosphere (DCA) storage have been developed to address this problem, allowing superficial scald and other postharvest disorders to be controlled more effectively^1,2^.

The optimal levels of O_2_ in controlled atmosphere and ultra-low oxygen storage are determined by trial and error, but must be above the anaerobic compensation point—the O_2_ level at which CO_2_ production is minimal^3^. In a dynamically-controlled atmosphere, the composition of the storage atmosphere is adjusted to the current physiological state of the fruit. When anaerobic stress is detected, the composition of the storage atmosphere is adjusted to maintain the O_2_ level as close as possible to the acceptable low oxygen level value of the product without dropping below it. The two most prominent types of dynamically-controlled atmosphere systems use measurements of chlorophyll-fluorescence and respiratory quotient as indicators of low oxygen level^2,4^. The common feature of dynamically controlled atmosphere and ultra-low oxygen storage is that the oxygen level in the storage atmosphere is kept above the anaerobic compensation point. Below the anaerobic compensation point the risk of severe quality loss increases due to anaerobic metabolism (fermentation) starting to dominate the metabolic activity of the fruit^3^.

However, oxidative stress is not always detrimental to fruit quality. Several studies have shown beneficial effects of short exposure of oxidative stress for stored fruit, such as better preservation, increased firmness, preservation of polyphenolic compounds, and reduced risk of postharvest disorders such as bitter pit and superficial scald in apples^1,2^. This led to alternative storage protocols such as initial low-oxygen stress storage (ILOS), consisting in the application of a single controlled O_2_ stress to the fruit or the repetition of multiple low oxygen stress periods, followed by storage in controlled atmosphere or ultra-low oxygen storage^1^.

The mechanism underlying the prevention of postharvest disorders by initial low-oxygen stress storage is still not completely clear. The induced oxidative stress results in ethanol accumulation that can reduce superficial scald^5,6^. The increase of ethanol production in low-oxygen stress-treated fruit has also been linked to lower production of reactive oxygen species^6^. Some studies suggest that antioxidant systems might play a role in providing protection against physiological disorders^7,8^. Low oxygen stress can also cause changes in the expression of genes related to the ripening and senescence of fruit, leading to a delay in the onset of these processes^9,10^. The activation of stress response pathways can help the fruit to adapt and survive under adverse conditions^11,12^.

In this study, apple fruit of three cultivars were exposed to hypoxic storage conditions of various lengths to induce anaerobiosis. The response of the apple fruit to short-term oxidative stress was evaluated collectively in terms of the composition and structure of plant cell wall pectic polysaccharides and the mechanics of cell wall material, and assessed by means of immunolabeling and atomic force microscopy (AFM). The aim was to assess the nano-scale effects of oxidative stress on apple fruit cell walls.

## Results

**Figure 1:**
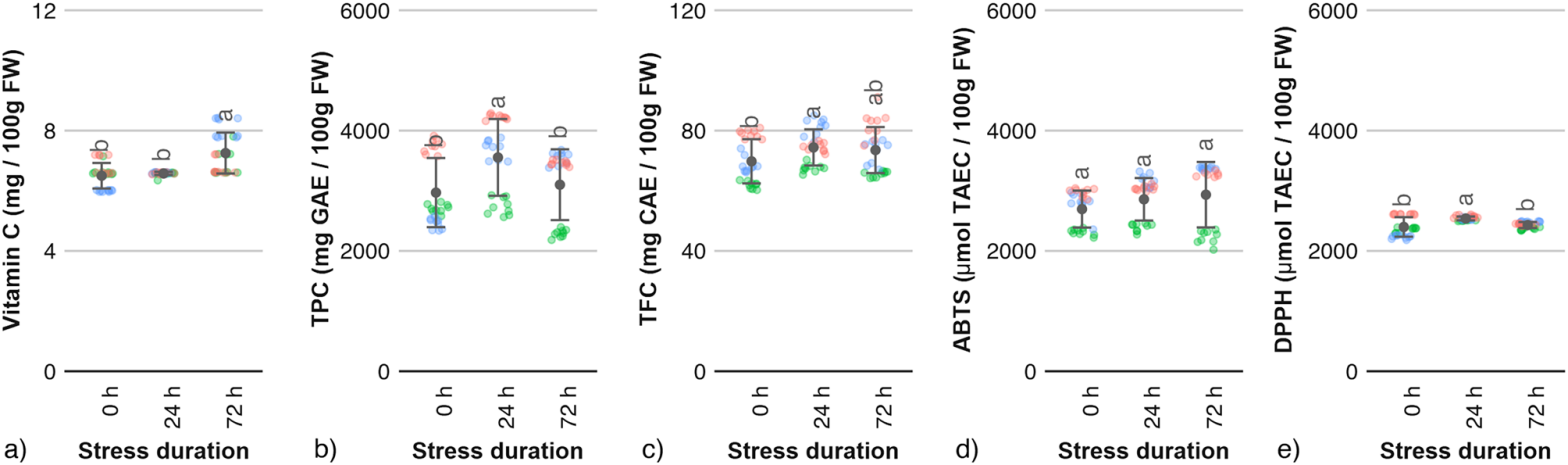
Chemical analysis of apple fruit material exposed to oxidative stress: (**a**) content of vitamin C, (**b**) total soluble polyphenols content, (**c**) total flavanol content, (**d**) antioxidant activity by means of ABTS, (**e**) antioxidant activity by means of DPPH. Data points show average values with standard deviations (points with bars) for all cultivars tested. Data points with different letter indices are significantly different (One-way ANOVA, Tukey HSD, *p* < 0.05, N = 27, three samples per each cultivar-treatment variant, three replicates per sample). Color-coded points indicate individual measurement for each apple cultivar: —Ligol cv., —Red Jonaprince cv., —Najdared cv.

The chemical analysis was carried out to assess apples’ antioxidant capacity and antioxidative activity (Fig. 1). Statistical analysis showed no significant effect of oxidative stress on either total soluble polyphenols content (TPC) or total flavanol content (TFC) levels (Fig. 1b,c). The amounts TFC after 72 h of stress were comparable with those found in a control sample, in case of all three cultivars.

**Figure 2:**
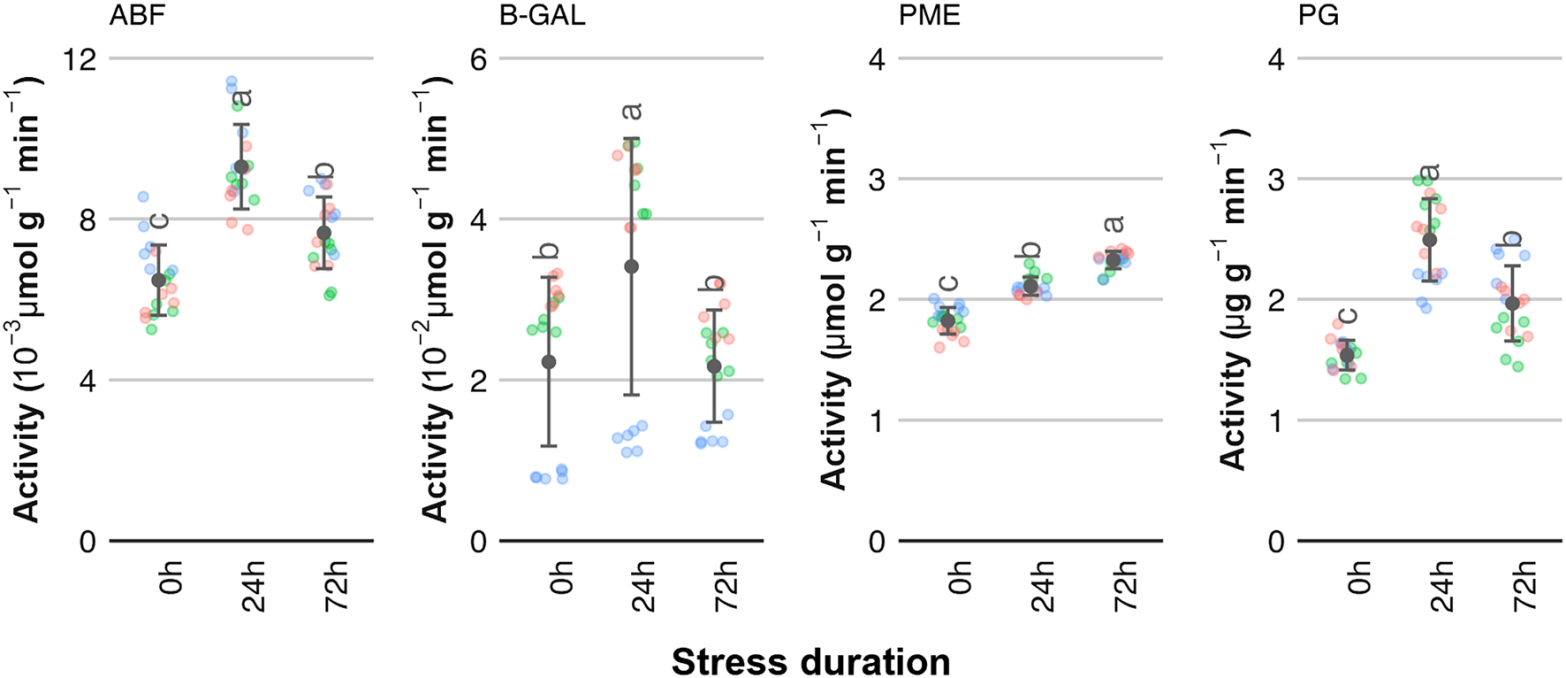
Enzyme activity measurements for apples subjected to oxidative stress of various durations. The activity of four enzymes was measured: α-L-arabinofuranosidase (ABF), β-galactosidase (B-GAL), pectin methylesterase (PME) and polygalacturonase (PG). Data points show average values with standard deviations (points with bars) for all cultivars tested. Data points with different letter indices are significantly different (One-way ANOVA, Tukey HSD, *p* < 0.05, N = 18, three samples per each cultivar-treatment variant, two replicates per sample). Color-coded points indicate individual measurement for each apple cultivar: —Ligol cv., —Red Jonaprince cv., —Najdared cv.

In case of Red Jonaprince and Ligol cv. the polyphenols content after 72 h of stress was at the same level or slightly lower compared with the control sample (Fig. 1b). In contrast, in Najadared cv. apples the polyphenols content increased after 24 h of stress and remained at the same level after 72 h of stress. In general, Ligol cv. apples showed lower TPC and TFC levels compared to the other two cultivars of fruit. The average vitamin C content increased after 72 h of oxidative stress, compared to a control sample and samples after 24 h of stress (Fig. 1a). However, this change was caused by an increase in vitamin C content for the apples of Najdared cv., while for the other cultivars the vitamin C level remained unchanged. The apples’ antioxidative activity, as indicated by ABTS and DPPH levels, showed a moderate response to the applied treatment. The ABTS levels increased steadily by a small amount with the duration of stress, showing a significant difference after 72 h of stress compared to a control sample for Najdared and Red Jonaprince cultivars. For the Ligol cv. apples, the ABTS values were lower compared to the other cultivars and remained unchanged after the stress was applied. At the same time, the DPPH levels after 72 h of stress remained similar to those found in a control sample. Exposure to oxidative stress resulted in significant changes in the activity of pectinolytic enzymes. The activities of α-l-arabinofuranosidase (ABF), pectin methylesterase (PME) and polygalacturonase (PG) increased along with duration of exposure of apple fruit to anaerobic stress (Fig. 2, ABF, PME and PG). The β-galactosidase (B-GAL) showed no significant increase in activity in apples after 72 h of stress exposure in case of Ligol and Red Jonaprince cv. apples, compared to control samples. A gradual increase in the activity of β-galactosidase was measured for the Najdared cv. apples, however the overall activity levels of B-GAL were significantly lower compared the other two apple cultivars. The pectin methylesterase showed gradual increase in activity with increasing stress duration. For α-l-arabinofuranosidase and polygalacturonase, the activity pattern showed peak activity after 24 h of stress and significantly higher activity after 72 h, compared to control samples.

**Figure 3:**
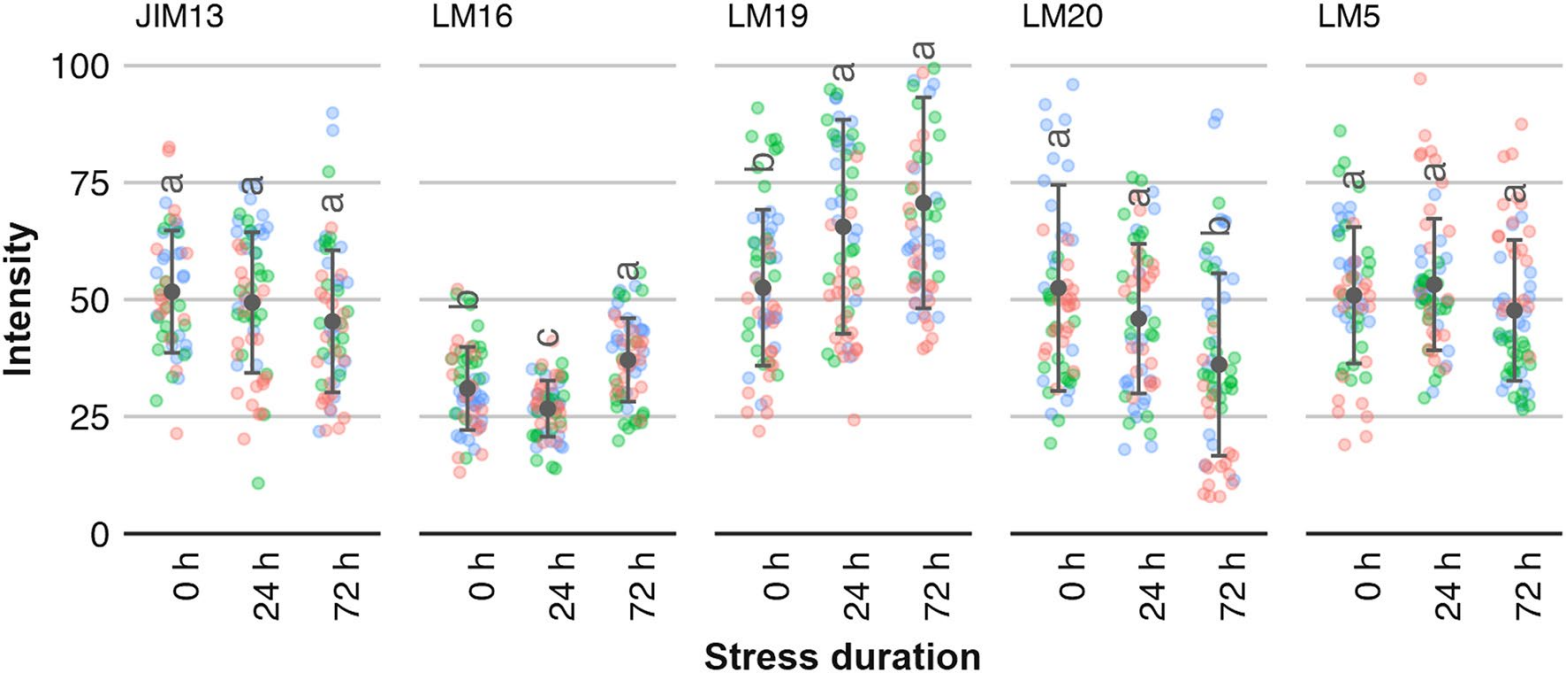
Average intensities from fluorescence images obtained using immunocytochemical labeling with JIM13 (arabinogalactan proteins), LM16 (galactosyl residue(s) on RGI backbones), LM19 (un-esterified homogalacturonan), LM20 (methyl esterified homogalacturonan), and LM5 (linear tetrasaccharide in (1–4)-β-D-galactans) antibodies. Data points show average values with standard deviations (points with bars) for all cultivars tested. Data points with different letter indices are significantly different (One-way ANOVA, Tukey HSD, p < 0.05, N = 60, five tissue samples per each cultivar-treatment variant, four slices/images per sample). Color-coded points indicate individual measurement for each apple cultivar: —Ligol cv., —Red Jonaprince cv., —Najdared cv.

The stress-induced activity levels of enzymes were reflected by the pectic polysaccharides content indicated by fluorescence images obtained using immunocytochemical labeling (Fig. 3). The amount of detected JIM13 and LM5 antibodies, specific to arabinogalactan proteins and linear tetrasaccharide in (1–4)-β-D-galactans, respectively, showed no significant changes with oxidative stress duration. The amount of the RGI-specific LM16 antibody initially decreased (after 24 h of stress), to finally increase (after 72 h) above the level reported for control samples. Although the changes were statistically significant, their extent was relatively small. The apparent trend can be considered random, and due to the natural variability of rhamnogalacturonan I in the sample.

An increase in PME and PG activity was followed by changes in cell wall pectin structure indicated by immunocytochemical labeling. As a result of higher PME activity, the amount of un-esterified homogalacturonan indicated by the LM19 antibody increased with the stress exposition time (Fig. 3). At the same time, the amount of detected methyl esterified homogalacturonan decreased, as indicated by the LM20 antibody. The microscopic images revealed different spatial deposition patterns of un-esterified and methyl esterified homogalacturonan domains in apple tissue (Fig. 3).

**Figure 4:**
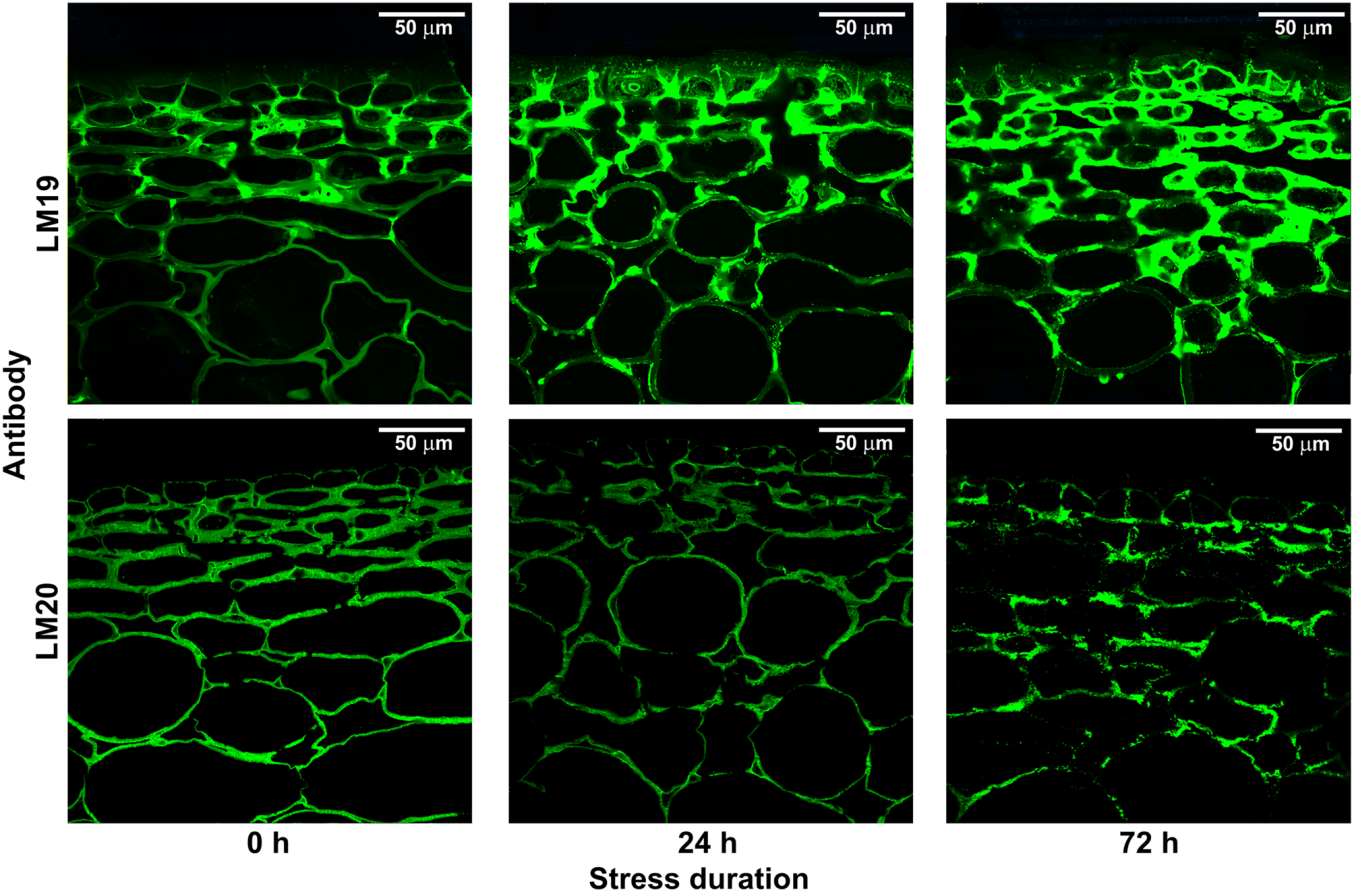
Example CLSM images of spatial deposition of LM19 (un-esterified homogalacturonan) and LM20 (methyl esterified homogalacturonan) antibodies in apple tissue (Ligol cv.) after 24 and 72 h of oxidative stress, compared with a control sample (0 h).

The LM20 measurements showed an equally distributed presence of methyl esterified homogalacturonan in the whole cross-section of apple tissue, with intensity gradually decreasing with stress duration (Fig. 4). For samples not subjected to oxidative stress the un-esterified homogalacturonan indicated by the LM19 antibody was localized in the areas between adjacent cells (i.e., at the corners of adjacent cells). A higher concentration of un-esterified homogalacturonan was observed in regions close to fruit skin. The stress induced reaction of the apple fruit resulted in an increase in the amount of un-esterified homogalacturonan, especially at the outline of cell walls (see Fig. S4 in supplementary materials) and in subepidermal regions of tissue. Similar observations were made for apple slices vacuum-impregnated with a pectin methylesterase, where immunolabeling showed that demethyl-esterification was more intense in the cell wall region lining intercellular spaces^13^.

**Figure 5:**
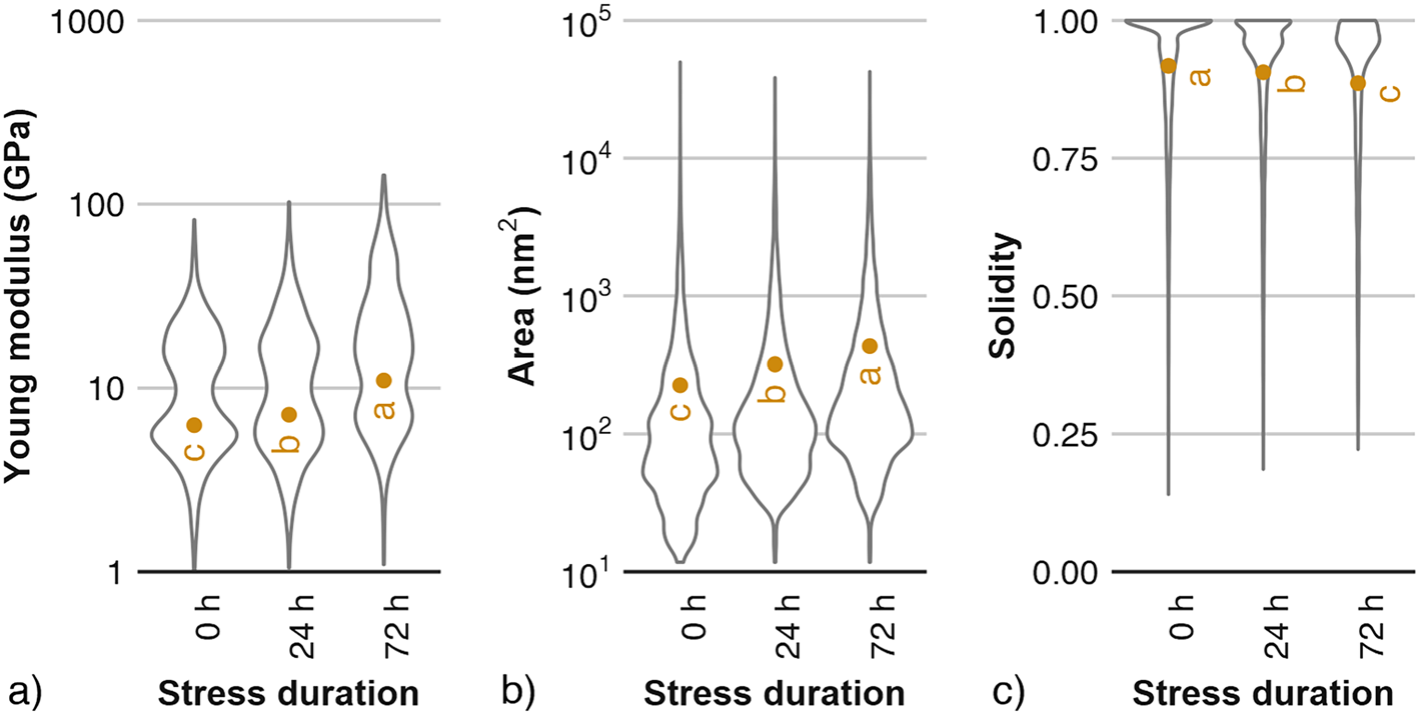
Nano-scale properties of apples subjected to oxidative stress of various durations: (**a**) Young modulus of dried cell wall material (N = 750, ten cell wall fragments per each cultivar-treatment variant, 25 individual sampling points per fragment), (**b**) average area of pectin molecules (N = 13,000), (**c**) average solidity of pectin molecules (N = 13,000). Data points show average values for all cultivars tested, while distributions of numeric data for treatments are depicted using density curves. Data points with different letter indices are significantly different (One-way ANOVA, Tukey HSD, *p* < 0.05).

**Figure 6:**
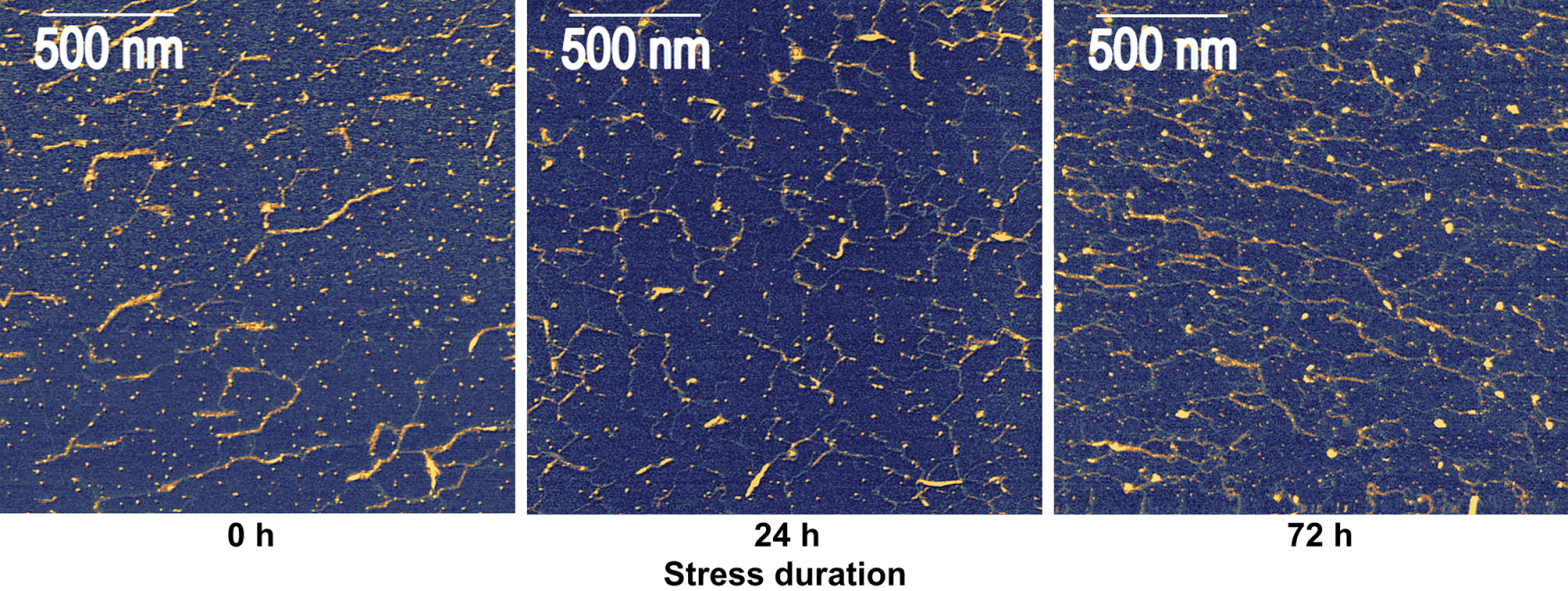
Selected AFM images of apple pectin (Jonaprince cv.) deposited on mica, extracted from fruit after 24 and 72 h of oxidative stress, compared with a control sample (0 h).

The nano-scale properties of dried cell wall material showed significant changes after exposure of fruit to oxidative stress (Fig. 5). The modulus of cell walls in a dry state measured by means of nano-indentation experiments increased from 6.2 GPa for a control sample, up to 10.9 GPa for apples kept for 72 h under oxidative stress. Along with increased stiffness of plant cell wall material, significant changes in pectin structure were observed (Figs. 5 and 6).

An increase in the area of individual pectic aggregates was observed with the increase in the duration of stress exposure for apple fruit (Figs. 5b and 6). After 72 h of stress, aggregates doubled their size on average compared to a control sample. The average solidity of aggregates decreased with stress duration (Fig. 5c), indicating that with stress exposure pectic structures became increasingly complex (i.e., consisted of more branches, were more entangled, and had longer and more curved shapes; see Fig. 6). AFM images show that the average solidity of pectic structures decreased from 0.91 to 0.88. The apparent decrease in solidity was low due to the fact that the values for this parameter were dominated by the presence of numerous small objects with solidity values close to 1.0. Nevertheless, the decrease was statistically significant and indicated the appearance of new, more complex structures, probably formed by the aggregation of smaller structures observed in a control sample.

## Discussion

The goal of applying initial oxidative stress is the preservation of apple fruit quality during long-term storage. The most commonly cited benefit of initial low oxygen stress followed by storage in air, controlled atmosphere or ultra-low oxygen storage is the reduction of the risk of occurrence of postharvest disorders such as bitter pit and superficial scald^1,2^. The exact mechanism of interaction is still not completely clear. It has been shown that low oxygen treatments may enhance antioxidant enzyme systems in plant tissues^8^ and antioxidant systems might play some role in protecting apple fruit against physiological disorders^7^. Short-term storage in an oxygen-reduced atmosphere has been shown to produce no beneficial effects on the content of polyphenolic compounds compared to conventional cold storage^14^. Higher content of glutathione, a bioactive compound capable of reactive oxygen species scavenging, has been reported after the low-oxygen treatment of apples^15^. As for flavonoids, long-term storage in ultra-low oxygen storage did not produce favorable effects compared to conventional storage techniques^16^. The researchers showed that for both ultra-low oxygen and conventional storage, flavonoid levels remain stable over time, as well as during shelf life. In this study the total content of both flavonoids and polyphenols in apple skin remained fairly constant regardless of the duration of oxidative stress applied. The exception was observed in case of Najdared cv. apples, which total polyphenol content increased with duration of the low-oxygen stress. A slight change in TPC was also observed after 24 h for Red Jonaprince cv., however after 72 h of stress exposition apples showed polyphenol levels comparable with the control sample. In general the observed changes were not consistent with the level of exposure to low-oxygen stress. Consistent changes were observed for total polyphenol levels of one apple cultivar only. Therefore it was concluded that the metabolic changes induced by oxidative stress in this study did not affect the content of these compounds in the fruit peel, probably due to relatively short periods of stress (up to 72 h, compared with 1 to 2 weeks in industrial applications). No change in antioxidant content was followed by a lack of change in the anti-oxidative activity of apples as expressed by ABTS and DPPH. Stanger et al.^17^ also found an absence of the effects of storage systems (various low oxygen storage methods) on the total anti-oxidative activity of apples. The obtained results put into question the possible role of antioxidative systems in protection against scald-like disorders in apples.

In contrast, stress-related metabolic changes had a significant impact on the enzymatic activity of apple fruit. The activities of pectin methylesterase and polygalacturonase, two main pectinolytic enzymes, increased along with the duration of oxidative stress. An increase in activity was also shown for α-l-arabinofuranosidase.

So far, the process of post-harvest fruit softening has mostly been associated with the action of two enzymes, PME and PG. Recently, more studies have suggested that fruit softening is a result of the synchronized cooperation of multiple enzymes, including hydrolase enzymes such as β-galactosidases and α-arabinofuranosidases^18^. ABF and B-GAL are considered late-ripening enzymes, the activities of which increase after fruit harvest as the fruit become over-ripe^19^. High ABF gene expression and enzyme activity levels have been associated with fruit mealiness^20^. Jones et al.^21^ has shown that the hydrolysis of cell wall arabinan prevents stomatal opening, suggesting that arabinans maintain flexibility in guard cell walls. The overall ABF activity levels reported in this study were higher than those found for unripe or fruit at harvest, but at the same time lower than for over-ripe fruit^19^. In plants, arabinose, the target molecule of ABF, occurs in pectic arabinan, rhamnogalacturonan II, arabinoxylan and arabinogalactan-protein^22^. However, in this study levels of arabinogalactan proteins indicated by the JIM13 antibody were constant and showed no significant changes related to oxidative stress.

Decrease in galactan content has been linked to fruit softening and changes in the firmness of several plant tissues^23^. However, in this study levels of B-GAL activity measured after 72 h of oxidative stress were the same as they were for the control samples. The amount of LM5, an antibody specific to linear tetrasaccharide in (1–4)-β-d-galactans, showed a similar lack of dependence on the duration of oxidative stress as JIM13 did, indicating that there were no structural changes in plant cell wall galactans.

The most prominent effects of oxidative stress were reported for PME and PG enzymes and the amounts of methyl esterified homogalacturonan and un-esterified homogalacturonan in plant tissues. The activities of both enzymes increased with stress duration, with PG reaching maximum activity after 24 h of stress and maintaining high activity levels after 72 h (Fig. 3). The amount of un-esterified homogalacturonan increased with stress duration, while at the same time the amount of methyl esterified homogalacturonan detected in apple tissue decreased (Fig. 4). It is believed that initial de-methylesterification of cell wall galacturonans by PME is required to make the cell wall matrix more accessible to other enzymes such as poly-galacturonanses^18^. Pectin methyl esterases catalyze the specific de-methylesterification of galacturonic acid residues comprising the HG chains. Due to the hydrolysis of homogalacturonan chains, methanol and protons are released into the apoplast, creating acidic pectin with negatively charged carboxyl groups.

In this study, the increase in PME and PG activity coincided with an increase in the stiffness of cell wall material (Fig. 5a). The obtained results also show a gradual increase in the size and complexity of aggregates created by pectic polysaccharides along with the increase in PME activity. An increase in stiffness of cell wall material could be explained by structural changes in the plant cell wall pectic matrix, especially in homogalacturonan structures. It has been shown that homogalacturonan rich regions of plants are considerably stiffer^24,25^. Other studies have linked homogalacturonan to cell adhesion^26^.

Among the possible mechanisms of cell wall stiffening, the calcium mediated bonding of homogalacturonan molecules provides a plausible explanation for observed changes in PCW mechanics and the structure of pectin. Limited oxygen access in plants was shown to create compound stress that affect many cellular processes^27^. Stress induces reactive oxygen species production leading in turn to increase in cytoplasmic calcium levels^28–30^. The increase in cytosolic calcium levels occurs through the release of calcium from the apoplast and/or cellular organelles^31^. In the presence of calcium ions the negatively charged carboxyl groups resulting from homogalacturonan de-methylesterificatin are known to form so-called “egg-box” structures, where at least eight residues along each of the participating homogalacturonan chains form a stable inter-chain association^32^. In solutions, this results in the formation of extended polysaccharide networks and strong gels^33^. In the case of plants, the Ca^2+^ interacts strongly with pectin, thereby increasing cell wall strength and fruit firmness^13^. The phenomenon of cell wall stiffening observed in this study coincides with these reports in the literature. It also provides a plausible explanation for the beneficial effects of short-term oxidative stress on stored apple fruit.

It was showed that calcium treatment improves post-harvest and post-storage firmness of apples^34^. The effect was particularly positive in the case of very soft apples due to greater extent of de-esterification of homogalacturonan. Moreover, an increase in cell-to-cell adhesion, as a result of gel formation by homogalacturonan in the presence of Ca^2+^, promoted crack propagation through cell walls instead of intercellular propagation, increasing the brittleness of the apple fruit. On the other hand, changes at the nanoscale are not necessarily easily reflected in the macroscale quality of the fruit. During postharvest storage of pears the cell wall Young’s modulus increased while firmness continued to decrease^35^. However, correlation analysis for the entire period of the experiment showed a lack of straightforward relation between the Young’s modulus of primary cell walls and fruit firmness.

Triggering the defense mechanism could also explain the observed effects of short term anaerobiosis on cell wall properties. PME activity can promote cell wall stiffening and the production of damage signals which are able to induce plant defense responses^36^. Pectin de-methylesterification has been shown to be required for the activation of plant immune responses, driven by oligogalacturonides perceived in *Arabidopsis* Pattern Recognition Receptors^37^. Increased PME activity is associated with the defense mechanisms of *Arabidopsis thaliana* and other plants when attacked by microbial pathogens^38^. In this context, triggering the defense mechanism could explain the beneficial effect of short term anaerobiosis prior to the cold storage of apples^1,2^. It has been shown that the application of low-oxygen stress for 10 days prior to the cold storage of Granny Smith apples can reduce superficial scald and bitter pit incidence during eight consecutive months of storage^1^. Also, low oxygen treatment of apple fruit has been shown to result in higher pectin methyl esterase gene expression, which is associated with greater firmness^1^. However, this mechanism should be considered as highly speculative, due to the lack of direct evidence.

The exposure of apple fruit from three cultivars to hypoxic storage conditions resulted in significant changes in the structure and composition of fruit cell walls. The short-term exposure to stress resulted in an increase in the activity of PME and PG enzymes and the remodeling of cell wall pectic polysaccharides. The amount of un-esterified homogalacturonan increased with the duration of stress exposure, while the amount of methyl esterified homogalacturonan decreased. It is concluded that the negatively charged carboxyl groups resulting from homogalacturonan de-methyl esterification led to the formation of Ca^2+^ mediated bonds between pectin molecules. This was observed as an increase in the size and complexity of extracted pectic polysaccharides. Structural changes in the cell wall also resulted in changes in its mechanical properties. The Young’s modulus of the cell wall material increased with the duration of exposure to oxidative stress.

## Materials and methods

### Sample material

Apples of ‘Najdared’, ‘Red Jonaprince’ and ‘Ligol’ cultivars (*Malus x domestica* Borkh) were used in this study. The apples were harvested within the optimum harvest window in 2022 and then stored in a cold room (controlled atmosphere, 2 °C) for 1 month prior to the experiments.

Batches of 20 kg of apples from each cultivar were then subjected to various durations of oxidative stress. Oxidative stress was induced by replacing the storage atmosphere with nitrogen. Detection of oxidative stress was carried out using a commercial apparatus based on the phenomenon of chlorophyll fluorescence DCA-CF (HarvestWatch™). Oxidative stress was maintained for periods of 24 and 72 h. Apples kept in a normal storage atmosphere served as a control sample. The effect of oxidative stress exposure was assessed for all cultivars collectively.

### Alcohol insoluble residue

The peel and core were removed from the apples, which were then cut into small pieces and homogenized with 96% ethyl alcohol. Next, the mixture of apple pulp and alcohol was stirred for 30 min and then filtered with vacuum pump using 0.45 µm nylon filters. The solid residue was mixed with alcohol and filtered again. This process was repeated until no sugars were detected in the apple pulp by means of the Dubois test^39^. The sugar-free sample was rinsed with 96% ethyl alcohol twice and acetone once, and then dried at 40 °C.

### Extraction of plant cell wall pectin (oxalate pectin)

The protocol proposed by Koubala et al.^40^ and Min et al.^41^, with some modifications, was used to extract oxalate pectins from apple cell wall material. Extraction was conducted in triplicate from each experimental variant. Thirty-five milliliter of 0.25% ammonium oxalate (Sigma-Aldrich Inc, St. Louis, USA) (pH = 4.6) was added to 1 g of cell wall material (CWM) and the mixture was incubated in 85 °C for 1 h. After incubation, the mixture was centrifuged (20,000 × g*,* 10 min) and the supernatant was collected. Three volumes of 96% ethanol were added to the supernatant and the obtained solution was incubated at 4 °C for 24 h. The solution was then centrifuged (20,000 × g, 10 min) and the supernatant was discarded. The precipitate, which contained pectin, was washed twice with 96% ethanol and then was dialyzed overnight using ZelluTrans/Roth^®^ dialysis membranes (MWCO 3500, Carl Roth GmbH + Co. KG, Karlsruhe, Germany). The last step was the freeze drying of the obtained oxalate pectins.

### Enzyme activity

The enzymatic activities of four pectinolytic enzymes, α-l-arabinofuranosidase (ABF), β-galactosidase (B-GAL), pectin methylesterase (PME) and polygalacturonase (PG) were determined according to Wei et al.^42^. The cell wall enzymes were extracted from 3 g of frozen and powdered fruit flesh mixed with 6 ml of cold 12 % polyethyleneglycol containing 0.2% sodium bisulphate. Then, the homogenate was centrifuged for 10 min at 21,000 × g and 4 °C and the pellets were washed in 4 °C aqueous 0.2% sodium bisulphate. The collected supernatant was mixed with an extraction solution (0.1 M sodium acetate, 100 mM sodium chloride, 2% mercaptoethanol and 5% polyvinylpyrrolidone in a 1:1:1:1 ratio). After 1 h of incubation at 37 °C, the solutions were centrifuged and the supernatants were used for enzymatic activity determination. Extracts were prepared in triplicate for each treatment-cultivar variant. The measurements of enzymatic activity were carried out in six replicates for each treatment-cultivar variant.

The activity of PG was determined using mixture of 0.2 ml of enzyme extract and 0.8 ml of 0.5 % polygalacturonic acid (in 50 mM sodium acetate buffer, pH = 5.2) incubated at 37 °C for 2 h. The incubation was followed by addition of 2 ml of 0.1 M borate buffer (pH = 9) and 0.3 ml of 1 M cyanoacetamide to the reaction mixture. Next, the mixture was placed in a water bath (at 100 °C) for 10 min. The absorbance at 276 nm was measured after the solution cooled down. Galacturonic acid was used as the standard. The enzymatic activity of PG was determined as µg g^−1^ min^−1^.

The activity of PME was determined using mixture of 4 ml of 1% (w/v) citrus pectin and 1 ml of enzyme extract. The mixture was titrated with 0.01 M sodium hydroxide to a pH value of 7.4 while incubating at 37 °C for 1 h. One unit of activity was defined as the amount of moles of sodium hydroxide required to titrate 1 g of the fruit pulp over a period of 10 min. The enzymatic activity of PME was determined as µmol g^−1^ min^−1^.

The activity of ABF and B-GAL was determined using mixture of 0.5 ml of the appropriate substrate (p-nitrophenyl-α-d-arabinofuranosidase and 3 mM p-nitrophenyl-β-d-galactopyranosidase for ABF and B-GAL, respectively) and 0.5 ml of 0.1 M sodium acetate (pH = 5.2), incubated at 40 °C for 10 min. Next, 0.5 ml of enzyme extract was added, and the mixture was incubated at 37 °C for 30 min. The reaction was stopped by adding 2 ml of 0.5 M sodium carbonate, and the absorbance at 276 nm was measured using a UV–Vis spectrophotometer (Genesys 10S, Thermo Scientific), with p-nitrophenyl used as the standard. The enzymatic activity of ABF and B-GAL was expressed as µmol of p-nitrophenyl released from 1 g of fruit pulp in 1 min (µmol g^−1^ min^−1^).

### Vitamin C content

Vitamin C content was determined using the titration method (PN-A-04019:1998, 1998). Fifty grams of fruit was blended in the presence of an extraction solution (2% oxalic acid) and filtered. Ten milliliters of the filtrate was immediately titrated with a solution of 2,6-dichlorophenol until a pale pink coloration was obtained. The vitamin C content was expressed as mg 100 g^−1^ FW. The measurements were carried out in nine replicates for each type of sample (cultivar-treatment variant).

### Antioxidant capacity

Apple peel extracts were obtained using the assay method proposed by Vieira et al.^44^. Extraction was carried out by adding 30 mL acetone-water (80:20 v/v) to 3 g of ground plant material and placed in an ultrasonic bath for 15 min at room temperature. Next, the samples were centrifuged at 15,000 rpm for 10 min at 5 °C and then supernatants were immediately tested. The measurements were carried out in nine replicates for each type of sample (cultivar-treatment variant).

#### Total soluble polyphenols content (TPC)

The TPC was measured using a modified Folin-Ciocalteu method^45^. Hundred µl of apple peel extract and 2.5 ml of water were mixed with 0.5 ml of Folin-Ciocalteu reagent. After 5 min of reaction, 1.5 ml 20% Na_2_CO_3_ was added, and mixture was made up to a volume of 10 ml with distilled water. The mixture was incubated for 120 min at room temperature before absorbance measurements were conducted at 765 nm using a UV–Vis spectrophotometer (Genesys 10S, Thermo Scientific). The polyphenol content of the apple peel was calculated against gallic acid standards and expressed as mg GAE 100 g^−1^ FW.

#### Total flavanol content (TFC)

The TFC content was measured using a modified p-dimethylaminocinnamaldehyde (DMACA) method^46^. 1 ml of apple peel extract was mixed with 5 ml of DMACA solution (0.1% in 1M HCl in MeOH) and vigorously shaken. After 10 minutes of incubation at room temperature, absorbance at 640 nm was measured using a UV–Vis spectrophotometer (Genesys 10S, Thermo Scientific). The flavanol content of the apple peel was calculated against catechin solution standards and expressed as mg CAE 100 g^−1^ FW.

### Antioxidant activity (AOA)

#### ABTS [2,2′-azinobis(3-ethylbynothiazoline-6-sulfonic acid)] assay

For the ABTS assay, the method described by Re et al.^47^ was used. The radical cation of ABTS (ABTS^•+^) was produced by the reaction of 7 mM ABTS with 2.45 mM K_2_S_2_O_8_. The reaction mixture was prepared 16 h prior to use and was kept in the dark at room temperature. Next, the ABTS^•+^ solution was diluted with ethanol to an absorbance of 0.700 ± 0.020 (t_0_) at 734 nm. Diluted ABTS^•+^ solution was added to 10 µl of apple peel extract, and after 7 min of reaction absorbance was measured (t_7_) using a UV–Vis spectrophotometer (Genesys 10S, Thermo Scientific). The percentage reduction of ABTS^·+^ was calculated as follows: $(100-\left[\frac{\mathrm{Absorbance\,}{t}_{7} }{\mathrm{Absorbance\,}{t}_{0}}\right]\times 100$). A standard curve of Trolox was prepared using the same procedure. The measurements were carried out in nine replicates for each type of sample (cultivar-treatment variant).

#### DPPH (1,1-diphenyl-2picrylhydrazyl) assay

A modified Brand-Williams et al.^48^ assay was used to measure the percentage reduction of DPPH^·^. Absorbance at 515 nm was measured for fresh daily prepared (t_0_) methanol DPPH^·^ solution (0.1 mM). Then, 0.1 ml of apple peel extracts was added to 2.9 ml of this solution and shaken with a vortex mixer. The sample’s absorbance at 515 nm was measured after 30 min (t_30_) in dark conditions and at room temperature using a UV–Vis spectrophotometer (Genesys 10S, Thermo Scientific). A standard curve of Trolox was prepared and then the percentage reduction of DPPH^·^ was calculated according to the equation: $(100-\left[\frac{\mathrm{Absorbance\,}{t}_{30} }{\mathrm{Absorbance\,}{t}_{0}}\right]\times 100)$. The measurements were carried out in nine replicates for each type of sample (cultivar-treatment variant).

### CLSM imaging with immunofluorescence technique

Sample preparation and immunocytochemical reaction was carried out according to the methods used in our previous investigations^49^. Immunocytochemical labeling was carried out with JIM13, LM16, LM19, LM20 and LM5 antibodies (Kerafast, Inc., USA), which are specific to arabinogalactan proteins, galactosyl residue(s) on RGI backbones, un-esterified homogalacturonan, methyl esterified homogalacturonan and linear tetrasaccharide in (1–4)-β-d-galactans, respectively.

Imaging was carried out using a confocal laser scanning microscope (CLSM) with a UPlanSApo 20x/0.40 objective (OLYMPUS FluoView300, Olympus Corporation, Tokyo, Japan). For each sample variant at least 20 images were captured. Imaging was carried out in the sub-epidermal region of the apple flesh, with apple skin visible in each image. The images were captured at a resolution of 1024 × 1024 pixels. Fluorescence intensity was expressed as the sum of the brightness of all pixels in each image divided by the area of the image (pixel brightness per μm^2^).

### Pectin imaging with atomic force microscope (AFM)

Twenty μl of 0.02 mg/ml oxalate pectin aqueous solution was uniformly distributed on a mica surface using a POLOS SPIN150i NPP spin coater (SPS-Europe B.V., Putten, the Netherlands). Samples were dried in a desiccator at room temperature overnight prior to AFM imaging. The imaging was carried out using a Multimode 8 AFM with a Nanoscope V controller (Bruker, Billerica, MA, USA), equipped with a silicon nitride cantilever SCANASYST-AIR-HR (Bruker, Billerica, MA, USA). The rectangular area of 2 by 2 μm was scanned at a resolution equal to 1024 × 1024 points. In total, 10 images of pectin on mica were collected for each variant of apple cultivar and treatment.

Basic image correction procedures were carried out using Gwyddion 2.52^50^. Segmentation by thresholding using a fixed level of height was carried out according to the procedure described by Szymańska-Chargot et al.^51^. The images were segmented and analyzed using a protocol developed using Matlab R2010a with the Image Analysis Toolpack (MathWorks Inc., Natick, Massachusetts, USA).

Images of individual pectic polysaccharides were characterized by two simple parameters, object area and solidity (the area fraction of the region as compared to its convex hull area), which describe the size and complexity of visible objects.

### CWM nano-indentation with atomic force microscope (AFM)

The cell wall material was prepared by grinding the AIR into powder. Next, the powder solution (0.1% w/w) was drop deposited onto microscope slides and left to dry for 72 hours. For each variant of sample, at least 250 nano-indentations were carried out. The Young modulus of cell wall material was estimated by means of the Hertz contact model^52^ using Nano Plot v1.1. software^53^. The nano-indentation was carried out using Bioscope Catalyst II (Bruker, Billerica, MA, USA), equipped with a silicon cantilever RTESPA-525 (Bruker, Billerica, MA, USA). The cantilever was calibrated prior to the experiments according to the protocols described by Zdunek and Kurenda^52^.

### Statistical analysis

Statistical tests (One-way ANOVA, Tukey HSD, *p* < 0.05) of the significance of differences between samples were performed using the ‘stats’ package (version 4.1.2.), which is a part of R^54^. The response of the apple fruit to short-term oxidative stress was evaluated collectively without distinguishing between cultivars. The aim was to show the overall effect of low oxygen stress on the apple fruit rather than the response of each cultivar individually. By analyzing the results without distinguishing between cultivars, I was possible to identify common trends or patterns that apply to apples as a whole.

### Ethics declarations

All methods were in accordance with the IUCN Policy Statement on Research Involving Species at Risk of Extinction and the Convention on the Trade in Endangered Species of Wild Fauna and Flora. The fruit was harvested from a commercial orchard located in Ostrowiec near Lowicz (52° 9′ 39.19″ N, 20° 3.40′ E).

### Supplementary Information

Supplementary Figures.

## Data Availability

The data supporting the findings of this study are available from the corresponding author upon request.
