# Supplementary material for: Apple metabolism under oxidative stress affects plant cell wall structure and mechanical properties
Source: Sci Rep. 2023 Aug 24;13:13879. doi: 10.1038/s41598-023-40782-6 (PMC10449782; doi:10.1038/s41598-023-40782-6)
Supplement: Supplementary file 1 — Supplementary Figures. [file 41598_2023_40782_MOESM1_ESM.docx]

**Supplementary materials:**

**Apple metabolism under oxidative stress affects plant cell wall structure and mechanical properties**

Piotr Mariusz Pieczywek^1^, Agata Leszczuk^1^, Magdalena Kurzyna-Szklarek^1^, Justyna Cybulska^1^, Zbigniew Jóźwiak^2^, Krzysztof Rutkowski^2^, Artur Zdunek^1^

^1^Institute of Agrophysics Polish Academy of Sciences, Lublin, Poland
^2^Institute of Horticulture - National Research Institute, Skierniewice, Poland


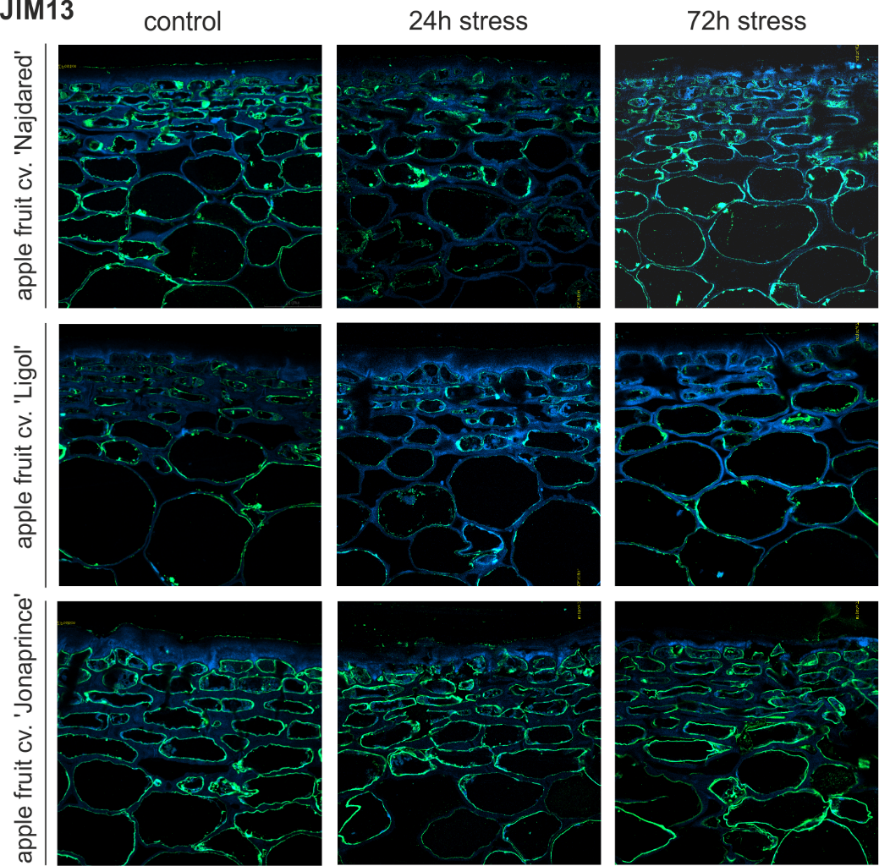


**Figure S1.** Immunocytochemical labeling of arabinogalactan proteins (AGPs) recognized by JIM3 antibodies in apple tissue after 24 and 72 hours of oxidative stress, compared with a control sample (0 hours). Antibody fluorescence is encoded using the green channel.


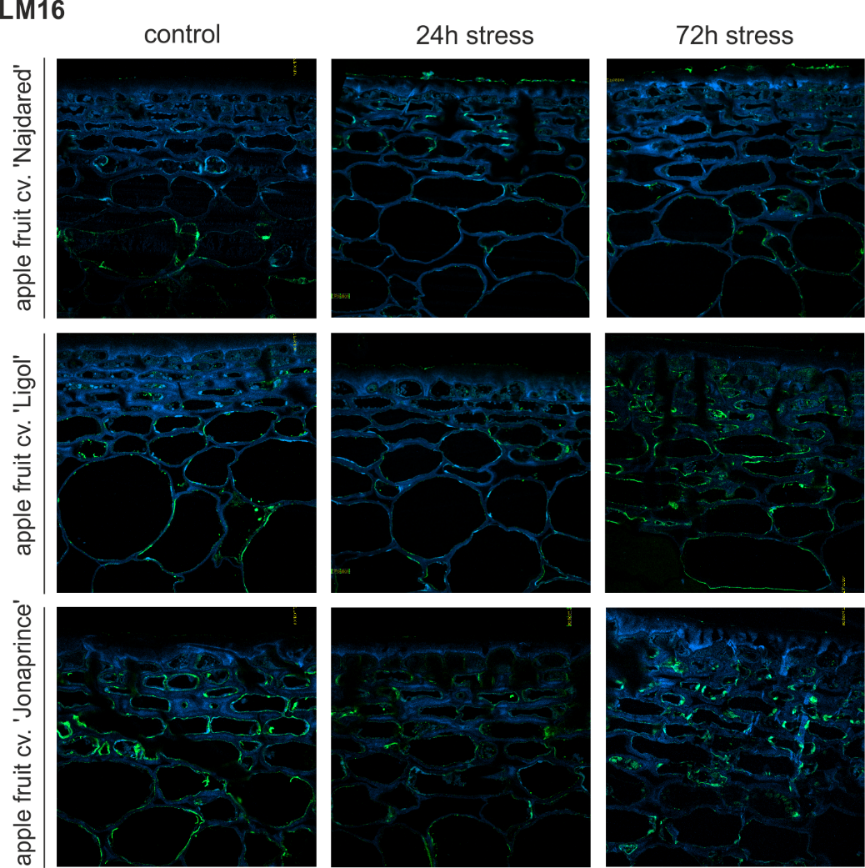


**Figure S2.** Immunocytochemical labeling of galactosyl residue(s) on RG backbones recognized by LM16 antibodies in apple tissue after 24 and 72 hours of oxidative stress, compared with a control sample (0 hours). Antibody fluorescence is encoded using the green channel.


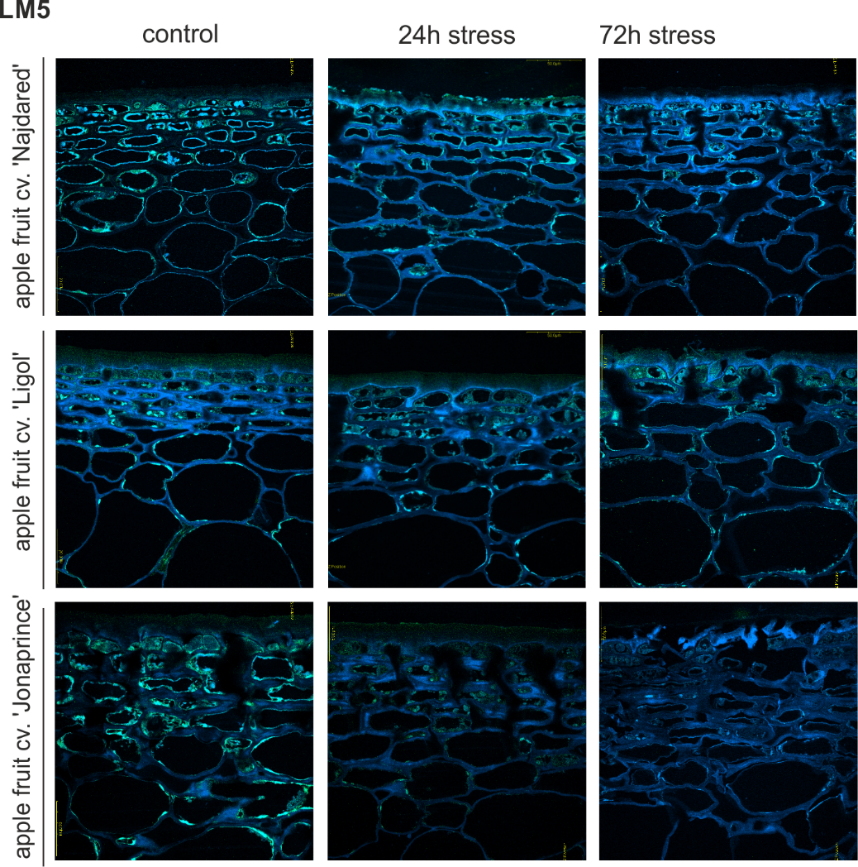


**Figure S3.** Immunocytochemical labeling of a linear tetrasaccharide in (1-4)-β-D-galactans recognized by LM5 antibodies in apple tissue after 24 and 72 hours of oxidative stress, compared with a control sample (0 hours). Antibody fluorescence is encoded using the green channel.


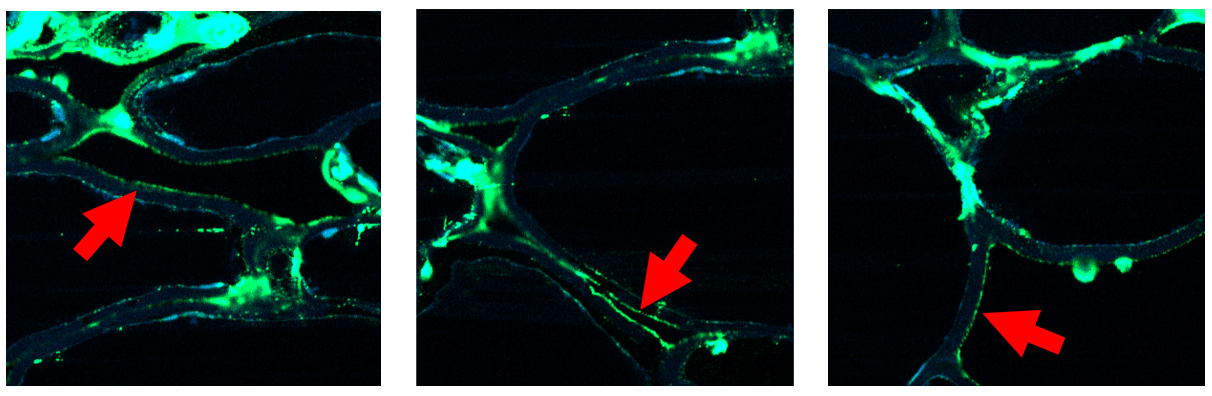


**Figure S4.** Examples detailed images of spatial deposition of LM19 (un-esterified homogalacturonan) showing an increase in the amount of un-esterified homogalacturonan at the outline of cell walls (red arrows). Antibody fluorescence is encoded using the green channel.
